# Adaptation to life after sport for retired athletes: A scoping review of existing reviews and programs

**DOI:** 10.1371/journal.pone.0291683

**Published:** 2023-09-21

**Authors:** Paula Voorheis, Michelle Silver, Josie Consonni

**Affiliations:** 1 Institute for Health Policy, Management, and Evaluation, University of Toronto, Toronto, ON, Canada; 2 Department of Health & Society, University of Toronto, Scarborough, ON, Canada; 3 College of Social and Applied Human Sciences, University of Guelph, Guelph, ON, Canada; University of Oviedo: Universidad de Oviedo, SPAIN

## Abstract

Retirement from sport is a life transition that has significant implications for athletes’ physical and mental health, as well as their social and professional development. Although extensive work has been done to review the retirement experiences of athletes, relatively less work has been done to examine and reflect on this expansive body of literature with a pragmatic aim of deciding what needs to happen to better support retiring athletes. This study used scoping review methodology to review current academic reviews, gray literature articles, and support programs on athletic retirement. This review followed the Joanna Briggs Institute reviewer’s manual guide on scoping reviews and adhered to the PRISMA-ScR checklist. Academic articles were identified from PubMed, Embase, Web of Science and Scopus. Gray literature articles and support programs were identified using advanced Google searches. This study identified 23 academic reviews, 44 gray literature articles, and 15 support programs. Generally, the results suggest that athletic retirement encompasses a drastic shift in identity, a loss of social networks, a lack of career ambitions, and potential risks to physical and mental health. While there was a gap in the academic literature regarding practical strategies to support retiring athletes, the gray literature suggests many creative ideas. Stepwise programming may be beneficial to help athletes: (a) make sense of their athletic experience and see retirement as an ongoing process; (b) develop a well-rounded sense of self identity and understand how to apply their unique skills and strengths in new ways; (3) gain control over their retirement transition by establishing a clear plan and adjusting to new routines and opportunities; and (4) normalize the transition experience by “living in the next” and building confidence in new life directions. Future research may benefit from developing and evaluating more programming to support athletes through the retirement transition.

## Introduction

### Background

Retirement from a high-performance sport is a life transition that has significant implications for physical and mental health, as well as social and professional development [1]. Athletic retirement tends to take place early in the life course, at a time when individuals are also at a critical point of engagement with their career trajectories and family planning pathways. High performance athletes possess tremendous drive and a willingness to exert themselves to their fullest potential [2]. The current literature demonstrates the numerous benefits of participation in sport including enhanced physical mobility and cognitive functioning [3–5]. Despite the many positive impacts of sport participation, retirement from sport can be a challenging transition with physical, social, emotional, and economic implications [6]. Specifically, retirement can be a difficult transition for athletes by prompting a recalibration of their identities away from athletic focus [7–10].

Reviews of the academic literature on athletic retirement have generated mixed findings regarding the impact of retirement from sport on mental and physical health [6, 11, 12]. The current literature generally links athletic retirement with depression [13, 14]. Some athletic retirement reviews point to mixed findings regarding cognitive health outcomes [15], though many reviews indicate that former athletes experience a higher occurrence of cognitive decline [16–19]. Nonetheless, there is also evidence that the adverse effects of athletic retirement decline over time as athletes adjust to new ways of life [6, 20]. Overall, evidence suggests that more works need to be done to understand the types of supports that would ameliorate the athletic retirement transition [19]. Furthermore, evidence suggests that it would be helpful to review and map the factors that help or hinder the retirement transition across a range of different sports and athlete sub-groups [1, 21–23].

Several approaches can be used to examine individuals’ retirement experiences, including approaches from the field of social gerontology where retirement is seen as a career transition that results in the termination of paid work [24–26], and a shift to a new career after a long period of employment in a different field [27–29]. Other theoretical work sees retirement as a transitional process [30–32] or even an event that occurs through several stages [33, 34] In sport science, athletic retirement has been conceptualized in a way that emphasizes emotional and social well-being [35–37], as well as non-athletic career planning [14, 38].

Regardless of the approach taken, there appears to have been extensive work done to review the retirement experiences of athletes in both academic and non-academic settings. Despite these important advancements, relatively less time has been taken to examine and reflect on the expansive body of literature, with a pragmatic aim of deciding what needs to happen next to better support retiring athletes. Despite numerous evidence syntheses, athletes still face physical, mental, and emotional struggles after retirement with a lack of appropriate supports. Though nearly every move an athlete makes is scrutinized, relatively little attention is paid to an athlete’s transition to life after sport. In light of the current state of the literature, this review presents a scoping review of the athlete retirement transition experience, focusing on existing reviews of the academic and gray literature, with a pragmatic aim to improve future practices.

### Research objectives and questions

The objective of this scoping review is to review other academic reviews, gray literature articles, and support programs that address athletes’ experience transiting into life after sport, with a pragmatic aim of identifying how athletes can be supported through the retirement process. To encompass a wide range of athlete experiences, this review includes both amateur and professional athletes. This review will address the following specific questions: (1) what are the main characteristics of academic and gray literature studies that review athletes’ transition experiences? (2) what does the current body of synthesized literature say about the key barriers and enablers to a successful athletic retirement transition? (3) what does the current body of synthesized literature say about the negative and positive impacts of athletic retirement? and (4) what does the current body of synthesized literature say about the programming needed to better support athletes’ transition into life after sport?

## Methods

### Study design

We followed the Joanna Briggs Institute reviewer’s manual guide on scoping reviews, with a focus on *other reviews* as our primary evidence source [39]. We wanted to compile evidence already synthesized on this topic to scope what is widely known about the research topic. Instead of focusing on reviewing methodological quality and evaluating effectiveness (i.e., as done in an umbrella review), we aimed to scope the literature more broadly with a pragmatic aim of informing future research and practice. Because of our pragmatic aims, we also included a gray literature review to identify (a) non-academic articles about athlete transitions, and (b) current programs that support athlete transitions. Our scoping review of reviews follows the PRISMA-ScR (Preferred Reporting Items for Systematic Reviews and Meta-Analyses Extension for Scoping Reviews) checklist [40].

### Search strategy

The search strategy was developed by the lead authors (PV and MS). To find relevant academic sources, a search of the databases PubMed, Embase, Web of Science and Scopus were conducted on July 23^rd^, 2022, with search terms related to athletes, retirement, and reviews. Results were limited to English. We ensured our search was specific enough to yield highly relevant reviews we knew we wanted to include. To find relevant gray literature sources, several targeted advanced Google searches were conducted between August and September 2022. The advanced Google searches included search terms related to athletes, retirement, and organizations that our research team identified as highly relevant (e.g., NCAA, USPORT, and the Canadian Olympic Committee). For pragmatic reasons, only the first 50 sources (i.e., approximately 5 pages of Google search results) were used for each advanced Google search. Our full search strategy can be viewed in **S1 Table.**

### Eligibility criteria

For our academic search, included papers were restricted to full-text, original, peer-reviewed evidence syntheses (e.g., systematic reviews, scoping reviews, rapid reviews). The reviews had to focus on athletes as their primary population (e.g., amateur or professional athletes). We decided to include amateur and professional athletes to encompass the wide range of athletes who experience the retirement transition process. The reviews also had to have a clear focus on athletes’ retirement or transition into life after competitive sport (e.g., athletes’ physical or psychosocial experiences upon retirement). Although we did not constrict our paper eligibility by the type of review, the review had to include a clear search strategy (e.g., a referenced database search and a report on the number of included articles). For our gray literature search, advanced google searches either identified: (a) programs that support athlete transitions, or (b) non-academic articles about athlete transitions. No restrictions were put on the date or location of included sources, although certain search terms focused on American and Canadian programs specifically.

### Evidence selection

Reviews from the academic database searches were handled using Covidence (Veritas Health Innovation Ltd) reference management software. Reviews were deduplicated and imported for a 2-level screening process. During level 1 screening, titles and abstracts were double screened by two reviewers (AR and LA) using the eligibility criteria. Publications with titles and abstracts not meeting the eligibility criteria were excluded. During level 2 screening, full-text papers that passed level 1 were screened by the same two reviewers (AR and LA). Studies that met the eligibility criteria were included for full data extraction. Discrepancies during level 1 and 2 screening were discussed over several meetings with final decisions on screening made by PV. Programs and articles from the gray literature search were handled using google sheets. One reviewer (JC) scanned the advanced google searches for relevant programs and articles using the eligibility criteria. The reviewer copied and pasted relevant programs and articles into a google sheet, which was then reviewed by PV. After discussion between JC and PV on the included programs and articles, the final gray literature source inclusion was finalized by PV.

### Data extraction and analysis

Data from the included academic reviews were extracted using a data extraction form that included information on reviews’ title, author, year, journal, review type, study objective, databases searched, date range searched, population limitations, setting limitations, number of papers included, paper types included (e.g., qualitative, quantitative, mixed methods), paper locations, paper populations (i.e., athlete gender, level, sport type, and retirement age), and the key thematic constructs identified in the review. The thematic information we extracted included information on (a) the barriers and enablers of athlete retirement transitions, and (b) the negative and positive outcomes of athletic retirement. Barriers to retirement transitions and negative outcomes after retirement were indicated using a negative text symbol (e.g., (-) high athletic identity, or (-) worsening anxiety and depression). Enablers and positive outcomes were indicated using a positive sign (e.g., (+) planned retirement, or (+) relief from pressure and expectation). The data was initially extracted by one reviewer (IH) and then reviewed and refined by the lead authors (MS and PV).

Data from included gray literature sources (i.e., programs and articles) were extracted using two different data extraction forms. Data from the gray literature *articles* were extracted using a data extraction form that included information on the title, author, host origination, organization mission, organization location, key themes about (a) athletes’ unique experiences, (b) athletes transition challenges and enablers, and (c) strategies or programs for improving the transition experience. Data from included gray literature *programs* were extracted using a data extraction form that included information on the title, author, host origination, organization mission, organization location, and the program’s offerings for athletes (e.g., a detailed description of what the program provides). The data was initially extracted by one reviewer (JC) and then reviewed and refined by the lead authors (MS and PV).

The lead author (PV) completed a descriptive analysis of the data collected from the included papers that aligned with the objectives of this review. For the academic sources, we start by presenting an overview of the review characteristics. We then present a summary of the major findings across the reviews depending on the review topic (i.e., (a) reviews that explore the general psychosocial experiences of retiring athletes, (b) reviews that specifically explore the cognitive and mental health experiences of retiring athletes, and (c) reviews that specifically explore the musculoskeletal and cardiovascular experiences of retiring athletes. For the gray literature sources, we first present an overview of the gray literature source characteristics. We then present a summary of the major themes across the gray literature sources, including summaries about (a) the unique experiences of athletes themselves, (b) athletes’ experiences with retirement, specifically, and (c) support programming for retiring athletes.

## Results

### Search results

**Fig 1:**
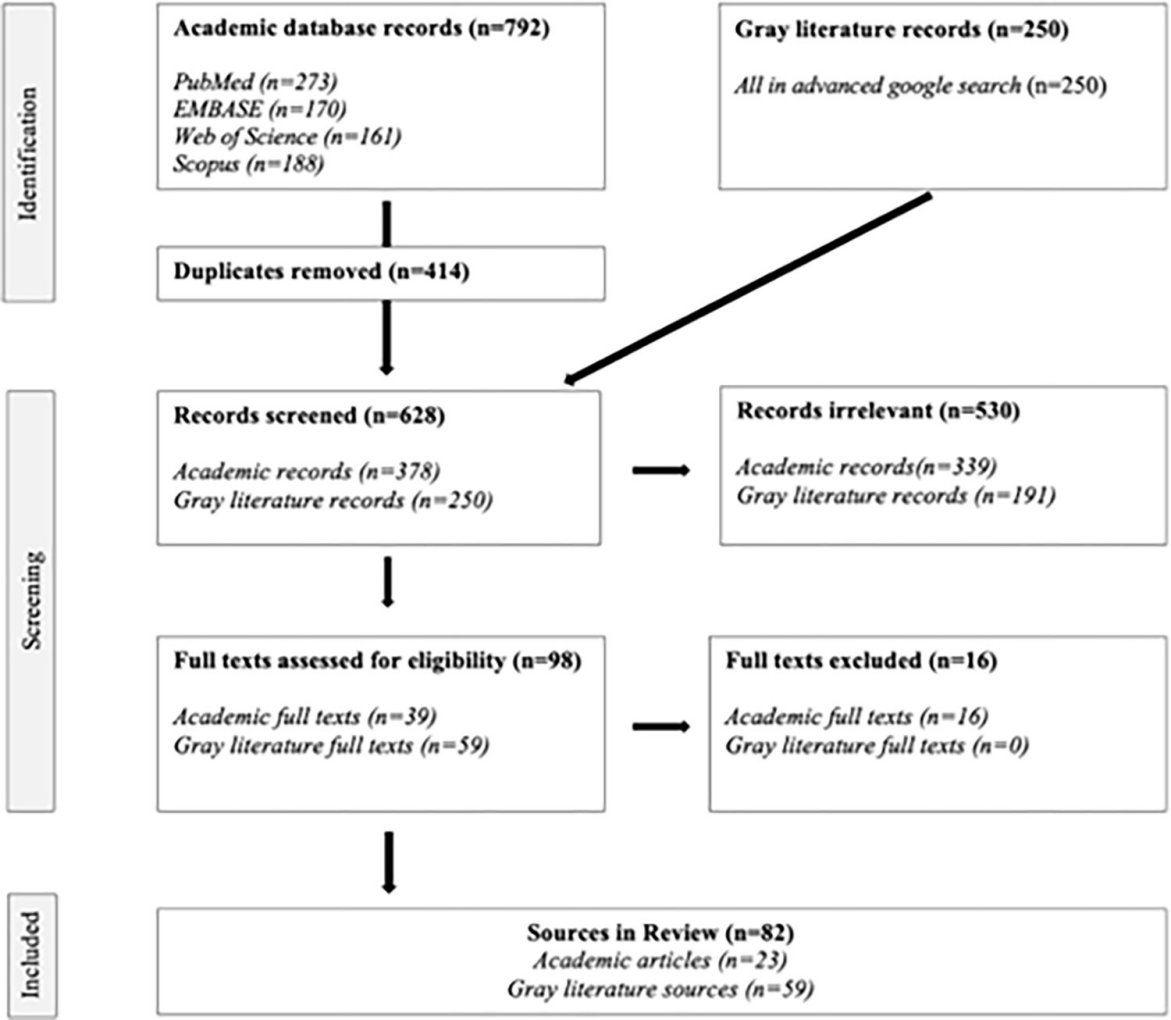
PRISMA-ScR flow diagram.

Fig 1 shows the PRISMA-ScR flow diagram illustrating the source selection process. A total of 792 and 250 sources were identified from the academic and gray literature searches respectively. After duplicates were removed, 628 records were screened based on their titles and abstracts, with 98 full text sources remaining for eligibility assessment. After a full text review, 82 sources were incorporated into the review, including 23 academic articles and 59 gray literature sources. Within the gray literature sources, 15 transition programs and 44 non-academic articles were identified.

### Results from academic literature

#### Review characteristics

We identified 23 academic reviews of literature examining the retirement transitions of athletes. A detailed description of the academic reviews included in this overarching review is provided in the **S2 Table.** All reviews were published in the last 10 years (between 2013–2022). In total, 10 reviews explored the general retirement experiences of athletes, 8 reviews explored athletic retirement with concussion, cognitive, and mental health concerns, and 5 reviews explored athletic retirement with musculoskeletal and cardiovascular health issues. The majority of the reviews were systematic reviews (n = 20), in addition there was a scoping review (n = 1), a critical review (n = 1), and a literature review (n = 1). Most reviews combined athletes from multiple different sports (n = 14); the remaining reviews focused on soccer players (n = 5), cricket players (n = 1), rugby players (n = 1), American football players (n = 1), and gymnasts (n = 1). Most reviews included athletes from multiple countries (n = 21), however, 2 reviews specifically focused on athletes in the United States. The number of articles examined in each review ranged from 4 articles to 219 articles, with the mode being 17 articles. Most reviews included male and female participants (n = 15), with the exception of reviews that focused on females only (n = 1), males only (n = 3), or did not report on gender (n = 4). Most reviews focused on quantitative studies (n = 16); however, several reviews included multiple study designs (n = 5). Only 1 review did not report the designs of the studies they included. The majority of reviews focused on professional and elite athletes (n = 11); several reviews featured sub-elite athletes (n = 6), such as club or high school athletes, and a substantial number of reviews did not report the level of the athletes in the studies included (n = 7).

#### General determinants and outcomes of athletic retirement

Among the 10 reviews that explored the general retirement experiences of athletes, we identified numerous barriers and facilitators that affected athletes’ ability to smoothly transition into life after sport. Barriers included: (1) having a high athletic identity; (2) high perfectionism and comparison; (3) athletic career dissatisfaction; (4) involuntary retirement (e.g., injury, contract, ability, or personal reasons); (5) amount of other life changes occurring at the same time as retirement; (6) despair about future career; (7) lack of coping strategies; (8) low social support; (9) lack of transition planning; (10) education and resource dissatisfaction; (11) previous concussions; (12) raising body mass index; and (13) onset of osteoarthritis. Facilitators included: (1) having a good sport-life balance (e.g., seeking other paths while still competing in sport); (2) positive self-perception and self-control; (3) athletic career satisfaction; (4) voluntariness of retirement; (5) gradual reduction of training; (6) length of time since retirement; (7) retirement planning; (8) support program involvement; (9) planned occupation after retirement (e.g., in sport or outside of sport); (10) educational attainment; (11) financial planning; (12) social support network; (13) continued relationships with coaching staff; (14) strong coping strategies (e.g., acceptance, reinterpretation; and (15) crisis preparedness.

In addition to barriers and facilitators that affected athletes’ retirement transition, we also identified numerous negative and positive impacts of athletic retirement itself. Negative impacts included: (1) worsening anxiety and depression; (2) feelings of burnout; (3) feelings of grief, loss, and void; (4) sleep disturbance and distress; (5) adverse alcohol use; (6) adverse smoking behaviours; (7) brain atrophy and dementia; (8) difficulty establishing new a lifestyle routine; (9) adverse nutrition behaviour; (10) disordered eating; (11) body dissatisfaction; (12) compensatory exercise; (13) high incidence of osteoarthritis incidence; (14) high incidence of join pain/back pain; and (15) high incidence of skin cancers. Positive impacts included: (1) relief from pressure and expectation; (2) high capacity for resilience and toughness; (3) greater or similar long-term quality of life; (4) greater capacity in certain mental proficiencies; and (5) difficulties with transition will fade over time.

#### Cognitive and mental health determinants and outcomes of athletic retirement

Among the 8 reviews that explored the relationship between athletic retirement and cognitive or mental health outcomes, several reviews examined and found that history of concussion during sport was a significant risk factor for cognitive impairment (e.g., memory, executive function, and psychomotor skills) and mental health issues among retired athletes. Only one review reported mixed findings about this association [15]. Most reviews found that retired athletes showed a high prevalence of cognitive impairment, anxiety, depression, and alcohol misuse compared to the general population. Only one review found that the prevalence of psychological distress was similar among retired athletes and the general population [41]. However, this review noted that risk factors such as a greater number of concussions, medical comorbidities, chronic pain, and low social support led retired athletes to have poorer outcomes [41]. Another review suggested that preparation for retirement and health exams were protective factors for retired athletes experiencing significant negative cognitive and mental health outcomes [42].

#### Musculoskeletal and cardiovascular determinants and outcomes of athletic retirement

Among the 5 reviews that explored the relationship between athletic retirement and musculoskeletal and cardiovascular health issues, 4 reviews specifically focused on osteoarthritis prevalence among retired athletes, while 1 review focused on cardiovascular disease risk [22]. Among the 4 reviews that focused on osteoarthritis prevalence among retired athletes, all these reviews found a higher prevalence of osteoarthritis (e.g., knee, hip, ankle) in retired athletes compared to the general population. The one review that focused on cardiovascular disease risk found that retired athletes shared a similar risk to that of the general population, however, athletes with a high body mass index were at an especially high risk [22].

### Results from the gray literature

#### Program and article characteristics

We identified 15 unique programs from our gray literature search that provide information about support for athletes through their athletic retirement transition. In addition, we identified 44 unique non-academic articles that describe and provide practical advice on athletic retirement transitions. Athlete retirement transition programs were based in different regions including, Canada (n = 7), the United Kingdom (n = 2), the United States (n = 2), Australia (n = 1), and South Africa (n = 1); while some programs had a clear global presence (n = 2). A full description of the programs and articles we identified in the gray literature search can be viewed in the **S3** and **S4 Tables.**

#### Unique experiences of athletes

Based on our analyses of the gray literature, 10 features emerged as unique aspects of the athlete experience: (1) living in a highly structured environment (e.g., following daily training routines); (2) living in a high pressure and stimulating environment (e.g., elevated attention and judgement); (3) athletics is experienced as a way of life (e.g., extraordinary time commitment); (4) high identity association with being an athlete (e.g., identity anchored to athletic achievements); (5) young career termination (e.g., feeling like a major part of your life has ended in your mid-20s or -30s); (6) high focus on body function and image (e.g., large focus on keeping body in peak performance); (7) experiencing injury and/or significant bodily changes (e.g., injury can be commonly associated with career derailment); (8) large personal sacrifices during their career (e.g., having to deprioritize time with family, friends, and romantic partners); (9) unique financial situations (e.g., making most of life earnings at a young age); and (10) gaining distinctive skillsets that can be transferable to other life circumstances (e.g., high-level teamwork, loyalty, punctuality).

#### Athletes’ experiences with retirement

In terms of the athletic retirement transition, 10 key challenges and 5 key opportunities emerged from the gray literature. Challenges include: (1) athletic termination as a crisis (e.g., feeling sudden grief, withdrawal, and emptiness); (2) athletic retirement as a long process, not a quick event (e.g., it might take a long time to adjust to a new way of life); (3) loss of identity (e.g., feeling decreased self-worth); (4) lack of purpose in day-to-day life (e.g., inability to make one’s own new daily routine); (5) lack of stimulation and psychological demands (e.g., nothing can provide the same thrill); (6) loss of significant relationships (e.g., change in social networks and loss of social support); (7) physical adaptations (e.g., change in weight, diet, and fitness); (8) financial concerns (e.g., worrying about maintaining lifestyle on a different income source); (9) mental health issues, (e.g., dealing with body dysmorphia, anxiety, depression); and (10) substance use concerns. Athletes’ transition opportunities relate to: (1) exploring new social networks and lifestyle opportunities (e.g., finding new hobbies and passions); (2) exploring other career interests (e.g., fulfilling life pursuits that had to be sidelined); (3) having the chance to unwind (e.g., embracing the relief from pressures around eating, drinking, and fitness); (4) the stamina and strength gained in athletics (e.g., high sense of resilience may be useful in building a new career); and (5) being psychologically disciplined and accountable (e.g., high sense of responsibility may be seen as advantageous in a new career).

Factors that influence whether an athlete has a positive or negative transition experience were found to be context dependent and related to issues such as: (1) voluntariness of retirement; (2) timing of retirement; (3) duration of the transition; (4) amount of other life changes during retirement; (5) positive or negative reflections on athletic career; (6) financial and career planning for retirement; (7) fitness and dietary planning for retirement; (8) personal brand built through athletics; (9) high athletic identity association; (10) ongoing injury issues; and (11) prioritization of retirement planning.

#### Support programming for retiring athletes

The primary themes that emerged in relation to athlete retirement transition supports include the deliverer of support, the receiver of support, the delivery mode of support, and the content of support. Regarding the deliverer of the support, many different groups provided athletes with support, including: (1) athletic organizations; (2) third-party organizations (e.g., organizations dedicated to helping athletes through retirement or mental health issues); (3) sports psychologists and counsellors; (4) family, friends, mentors, and peers; and (5) coaching staff and other team personnel (e.g., dieticians and trainers). Regarding the receiver of supports, many different groups also accepted support, including (1) athletes themselves; (2) coaching staff; (3) athletic organizations; and (4) parents, family, friends, and partners. Regarding the delivery mode of support, avenues tend to be resource-based (e.g., a physical or online items) or systems-based (e.g., a continuous support system established). Resource-based supports include: (1) resource repositories; (2) educational information; (3) employment lists; (4) extracurricular activities lists; (5) educational opportunities lists; (6) videos with education/advice; (7) podcasts sharing lived experiences; (8) speaker series sharing lived experiences; (9) social media stories; (10) assessment tools; (11) reflection workbooks; and (12) guided journaling. Systems-based provisions include: (1) person-to-person support (online or in-person); (2) email or text-based support; (3) stepwise multicomponent programming; (4) team-integrated programming; (5) small group workshops; (6) membership to a community network; and (7) conferences or retreats. Regarding the content of support, the following issues were addressed: (1) personal wellbeing; (2) identity shifting; (3) mental health; (4) social networks; (5) personal relationships; (6) career building; (7) educational development; (8) physical health; (9) diet and exercise; (10) financial planning; (11) stories about others lived experiences; (12) education about transitioning; (13) personalized sports psychology; and (14) stepwise programming.

Stepwise programming tended to include multifaceted interventions that featured the following steps: (1) come to terms with their retirement and see the retirement transition as a process; (2) develop a well-rounded sense of self identity and understand how to apply their unique skills and strengths in a new setting; (3) gain control over their retirement transition by establishing a future plan and adjusting to new routines and opportunities; and (4) normalize the transition experience by “living in the next” and building confidence in new life direction. The contents of these steps have been fully detailed in **S5 Table**.

## Discussion

This paper presents a scoping review of the synthesized academic and gray literature regarding athlete retirement transitions. Our analyses of review articles from the academic literature illustrate that possessing a *high athletic identity* is consistently associated with difficulties in transitioning into life after sport. Among elite athletes, it is not uncommon for their social networks, career ambitions, physical goals, psychological stimulation, and sense of self-worth to be distinctly tied to their sport [5, 36]. It is thus understandable that when an athlete’s competitive sporting life concludes, not only do they lose their sport, but athletes also substantially alter and may lose a core aspect of their personal identity [43, 44]. The literature also points to a clear need for athletic organizations to recognize this challenge to athletes’ personal identity and to provide supports to ameliorate athletes’ retirement transitions [10, 45].

In addition to the challenge of athletic identity, the academic literature highlighted an important threat for athletes who *ended their career on a low* (i.e., who were dissatisfied with their performance or were forced to retire due to injury, finances, skill, or personal issues). In line with prior research [41, 46, 47], it is clear that athletes whose career terminations were characterized by regret and loss were particularly prone to difficulties with their retirement transition; an abrupt ending to an athlete’s sporting career without purposeful planning can leave athletes feeling lost. In particular, *strong athletic identity* combined with *a traumatic career termination* puts athletes in a very difficult scenario early in their lives [48]. In line with prior research [49–51], this paper suggests athletes can greatly benefit from learning how to apply the skills developed as athletes to formulate new pathways in life outside of competitive sport.

Although identity loss, career regret, and abrupt lifestyle modifications were common conditions among retiring athletes, several key factors emerged across the academic literature as enablers of positive retirement transitions. The first notable enabler was that athletes who create a *healthy sport-life balance* over the course of their athletic career can better transition into life after sport. Developing interests outside of sport and making time to develop contingency plans for life after sport helped ameliorate the transition. A second notable enabler was that athletes who can *gain a sense of perceived control over their athletic termination*, and *gradually reduced their athletic participation*, are able to transition better into retirement. Although supporting athletes to maintain balance, plan for their retirement, and gradually detrain themselves may seem counterintuitive to sports organizations who want the most out of their current athletes, our paper indicates that athletes who plan for retirement may actually perform better while still competing.

While much attention has been placed on the need to enhance athlete mental health and wellbeing [52, 53], less consideration has been given to whether athlete retirement planning should be a key part of mental health programming. Findings from this review support the need to ease the pressure of the “sports-or-nothing” mentality [54, 55], thus emphasizing the notion of developing individuals as “more than an athlete”. The “more than an athlete” mantra has been widely used across the athletic community to push athletes to discover new ways to be their best selves, introducing other potential passions for athletes to follow. The evidence from this review suggests that athletes ought to be encouraged to develop a well-rounded sense of self, which is not exclusively focused on their athletic competition, but embraces their unique skillsets to connect with the world in distinct ways.

Across the synthesized literature is a consensus that retiring athletes are at a particularly high risk of experiencing *cognitive impairment*, *mental health issues*, *and osteoarthritis complications* [6, 7, 36]. These clinical findings suggest that not only should athlete retirement programming focus on helping athletes prepare for subsequent career transitions and make sense of their athletic career in relation to their personal identity, but they should also include interventions to address these specific health issues. For instance, mental health and physical health screening programs may be able to better identify retiring athletes who need specialized support, medical equipment, nutritional counseling, or rehabilitative therapy.

Despite indicating that retirement planning facilitated a better transition to athletic retirement, a clear gap in the academic literature exists with regard to how to develop and implement specific strategies for retirement planning based on sport and level. Fortunately, much of the gray literature focuses on these specific details, suggesting a multitude of creative ideas for how to better support athletes transition into life after sport. What is clear across the gray literature is that athletes need *a wide range of* supports from different sources in order to address different needs. Stepwise programming, where athletes work through a series of ongoing interventions to prepare for a retirement transition, emerged as a robust enabler of a positive transition experience. Gradual, longstanding, embedded investments into retirement planning may be highly advantageous for improving athlete retirement transition outcomes. Researchers and practitioners may deeply benefit from investigating the mechanisms that best support the development of a well-rounded sense of self throughout an individual’s athletic career and through the retirement transition experience.

### Strengths, limitations, and directions for future research

This review is the first of its kind to examine existing review articles from academic literature as well as current evidence from gray literature on retirement from sport. Limitations of this review include that we only included studies published in English and therefore may have excluded potential studies published in a different language. In an effort to be inclusive of the existing literature on athletic retirement, our review incorporates reviews of studies that combine different types of sport, multiple geographic regions, individuals of varying gender, and combines data on professional as well amateur athletes. Retirement experiences are likely to differ based on factors like these. Future research may benefit from focusing on developing and evaluating retirement support interventions based on characteristics of the individual athlete, the sport, as well as regional variations and other contextual factors.

## Conclusion

This review scopes the current synthesized academic and gray literature on athlete retirement transitions, with a pragmatic focus on how retiring athletes can be better supported. We identified and examined 23 academic reviews of literature examining the retirement transitions of athletes, 15 unique programs from gray literature that aimed to provide information about support to athletes through their athletic retirement transition experience, and 44 distinct non-academic articles that described and provided practical advice on the athletic retirement transition. The literature illustrates that feelings of loss, identity crisis, career regret, and difficulties with abrupt lifestyle modifications are common experiences in an athlete’s retirement transition. While the academic review literature presented a gap in evaluating programs that aim to ameliorate athletic retirement, the gray literature provided multiple creative ideas for how to improve programming to support athletes’ transition into life beyond sport.

## Supporting information

S1 Checklist (PDF)

S1 Table Search strategy. (DOCX)

S2 Table Included academic reviews. (DOCX)

S3 Table Gray literature articles. (DOCX)

S4 Table Gray literature programs. (DOCX)

S5 Table Stepwise programming content. (DOCX)
