# Supplementary material for: Adaptation to life after sport for retired athletes: A scoping review of existing reviews and programs
Source: PLoS One. 2023 Sep 21;18(9):e0291683. doi: 10.1371/journal.pone.0291683 (PMC10513329; doi:10.1371/journal.pone.0291683)
Supplement: S1 Table — (DOCX) [file pone.0291683.s002.docx]

**Supplementary Appendix 1. Search Strategy**

***Academic database search strategy (July 23^rd^, 2022)***

(athl* OR sport*) AND (retir* OR former OR transition*) AND ("systematic review" OR "scoping review" OR "narrative review" OR "meta-analysis" OR "umbrella review" OR "rapid review" OR "meta-ethnography" OR "interpretive synthesis" OR "realist review")

Limit to English Language

***Gray literature search (August – September 2022)***

*1. Advanced Google Search-* Use the following link to conduct a targeted google search: https://www.google.ca/advanced_search

ALL WORDS: athlete AND transition. ANY OF THESE WORDS: retirement OR career OR mental health OR counselling OR coaching

ALL WORDS: athlete AND retirement. ANY OF THESE WORDS: transition OR career OR mental health OR counselling OR coaching

*2. Targeted Organizational Search* - Search for offerings by specific organizations such as NCAA,

SEARCH: Canadian Olympic Committee AND Athlete Retirement Transition

SEARCH: NCAA AND Athlete Retirement Transition

SEARCH: USPORT AND Athlete Retirement Transition
