# Supplementary material for: Adaptation to life after sport for retired athletes: A scoping review of existing reviews and programs
Source: PLoS One. 2023 Sep 21;18(9):e0291683. doi: 10.1371/journal.pone.0291683 (PMC10513329; doi:10.1371/journal.pone.0291683)
Supplement: S2 Table — (DOCX) [file pone.0291683.s003.docx]

**Supplementary Appendix 2. Included Academic Reviews**

| **Title** | **Author, Year** | **Journal** | **Type of synthesis** | **# of papers included** | **Types of papers included** | **Settings included** | **Populations included** | **Key thematic constructs** |
| --- | --- | --- | --- | --- | --- | --- | --- | --- |
| **REVIEWS ABOUT: General Retirement Experiences and Implications (n=10)** | | | | | | | | |
| Retirement of professional soccer players - A systematic review from social sciences perspectives | Barth, 2021 | J Sports Sci | Systematic review | 17 | Quantitative (12), Qualitative (4), Mixed (1) | Great Britain (7), International (4), Ireland (2), Denmark (1), Portugal (1) Other (2) | **Gender:** male (16), combined (1)// **Level:** All professional (17) // **Sport type:** All soccer (17)// **Ages (at retirement):** 27-40 (10), not reported (7) | **Barriers/ facilitators of athlete transition**: (+/-) Different reasons for retirement (personal, injury, career change, contract end, declining ability), (-) Low planning for retirement, (+) High educational level led to better career attainment after retirement, (-) High dissatisfaction with education/welfare resources before retirement. **Impact of** **Retirement on health/ wellbeing outcomes:** (-) Worsening anxiety/depression, burnout, distress, sleeping disturbance, (-) Adverse nutrition behaviour, (-) Adverse alcohol, (-) Adverse smoking, (-) Difficulty re-defining identity, (-) Difficulty establishing routine with lack of clear focus, direction and timetables. (+) More relief from pressure and expectation, (+) Difficulties at the beginning of retirement fade in the long-term, |
| Retired Athletes and the Intersection of Food and Body: A Systematic Literature Review Exploring Compensatory Behaviours and Body Change | Buckley, 2019 | Nutrients | Systematic review | 16 | Quantitative (8), Qualitative (4), Mixed (4) | USA (8), France (3), International (3), Canada (1), Netherlands (1) | **Gender:** Male (3), Female (7), Mixed (6)// **Level:** unspecified// **Sports:** Gymnastics (5), Swimming (4), unspecified various (4), Soccer (2), Olympic sports (1), Rugby (1), NCAA sports (1)// **Ages (at retirement):** 16-26 (8), 27-40 (6), unknown (2) | **Barriers/ facilitators of athlete transition:** (-) Athletic career dissatisfaction, (-) retirement due to injury, (-) inadequate social support, (-) strong identity as an athlete after retirement, (-) learned culture of competition, perfectionism, and comparison. **Impact of retirement on health/ wellbeing outcomes:** (-) Body dissatisfaction and grief, (-) Disordered eating and compensatory exercise, (+) Difficulties at beginning of retirement fade over time, |
| The Relationship Between Cricket Participation, Health, and Wellbeing: A Systematic Scoping Review. | Bullock, 2022 | Int J Sports Med | Systematic review | 219 | Quantitative (119), Qualitative (6), Case Study (33), Systematic Review (15), Clinical Review (15), Other (31) | Australia (39 %), the United Kingdom (28 %), South Africa (11 %), India (8 %), New Zealand (5 %), the United States (4 %), Sri Lanka (2 %), Pakistan (1 %), Malaysia (1 %) and 1 ( < 1 %) were from Bangladesh, Nepal, Netherlands, and Singapore | **Gender:** Male (93%), Female (4%), Mixed (3%)// **Level:** 59% multiple levels, 26% national/international, 12% high school, 3% state// **Sports:** All cricket// **Ages (at retirement):** unknown. | **Impact of retirement on health/ wellbeing outcomes**: (-) Greater odds of osteoarthritis, (-) Greater odds of skin cancers, (-) Greater odds of alcohol abuse, (+) High capability of resilience, attitude, (+) Greater long term quality of life |
| Sports career termination in football players: Systematic review | Carapinheira, 2018 | South American journal of exercise and sport psychology | Systematic review | 8 | Not reported in original text. | UK (2), Brazil (1) Ireland (1), Kenya (1), Italy (1), International (2) | **Gender:** not reported// **Level:** all professional// **Sports:** All soccer// **Ages (at retirement):** 16-26 (1), 27-40 (4), Unknown (3) | **Barriers/ facilitators of athlete transition:** (+/-)Type of retirement as voluntary vs. involuntary, (+/-) Causes of retirement including age, other career interests, decreased physical ability, lack of incentives, family, injury, (+/-) Strong athletic identity, (-) Despair about future employment, (+) Occupations in sport after retirement, (+) Professional Footballers Association, (+) Family and friend support. **Impact of retirement on health/ wellbeing outcomes:** Mental health issues including sadness, anxiety, depression, (-) Alcohol abuse (-) Harmful food habits |
| Health conditions among retired professional footballers: a scoping review. | Carmody, 2022 | BMJ Open Sport Exerc Med | Scoping review | 41 | All quantitative (41) | Brazil(1), Greece (3), Switzerland(1), UK (9) , France(1), International (7), Finland (3), Denmark(2), Germany (5), Slovenia(1), Sweden (2), China(1), Turkey(1), Norway (2), Italy (1), Netherlands (1) | **Gender:** Male (36), Female (4), Mixed (1)// **Level:** All professional; **Sports:** All soccer// **Ages (at retirement):** Not reported | **Impact of retirement on health/ wellbeing outcomes:** (-) High osteoarthritis, (-) High joint pain, (-) High spinal issues and back pain, (-) High depression and anxiety, (-) High sleep disturbance, (-) Alcohol abuse, (-) Poor nutrition, (-) Smoking, (-) Dementia and brain atrophy |
| Quality of Life and Life Satisfaction in Former Athletes: A Systematic Review and Meta-Analysis | Fillbay, 2019 | Sports Med | Systematic review (and meta-analysis) | 17 | All quantitative (17) | Brazil (3), Finland (1), UK (2), International (1), USA (9), Australia (1 | **Gender:** Male (9), Mixed (7), Not reported (1)// **Level:** Professional (6) Olympic/International (2), University/NCAA (8), Elite (1)// **Sport:** Soccer (4), Multiple (8), Rugby (1), American football (4), Basketball (1)// **Age of retirement:** Not reported (8),16-26 (3), 27-40 (6) | **Impact of retirement on health/ wellbeing outcomes**: (+) On average retired athletes report similar physical QoL scores to the average population. (+) On average, retired athletes reported BETTER mental aspects of QoL compared to the general population. **Barriers/ facilitators of athlete transition**: There are a few variations to the above: (-) Higher BMI and poorer QoL post-retirement, (-) Involuntary retirement and poorer QoL post-retirement, (-) Higher contact sports and poorer QoL post-retirement, (-) High concussion rates and poorer QoL post-retirement, (-) Osteoarthritis onset and poorer QoL post-retirement. |
| Investigating Elite End-of-Athletic-Career Transition: A Systematic Review | Knights, 2016 | Journal of applied sport psychology | Systematic review | 10 | All quantitative (10) | International (1), Greece (1), Spain (1), France (2), Canada (1), Slovenia (1), Australia (2), Sweden (1), USA (1), UK (1) | **Gender:** Male (1), Mixed (7), not reported (2)// **Level:** College (1), National/International (8), Olympic (2)// **Sport:** Not reported// **Age of retirement:** All below 40. | **Barriers/ facilitators of athlete transition:** (+) Planned retirement led to higher cognitive, emotional, and behavioural readiness, (+) Non sport career plans, (+) Achieved sporting goals, (-) Involuntary retirement including injury, (-) High levels of athletic identity, (+) Educational status, (+) Coping strategies including acceptance, reinterpretation, and planning. I**mpact of retirement on health/ wellbeing outcomes:** (-) Emotional difficulties with loss and a void. |
| Are NCAA Division I Athletes Prepared for End-of-Athletic-Career Transition? A Literature Review | Miller, 2018 | Journal of Evidence-Informed Social Work | Literature review | 14 | Both quantitative and qualitative (numbers not reported) | United States (14) | Not reported in original text. | **Barriers/ facilitators of athlete transition**: (+) Life development intervention focused on career transition, (-) Strong athletic identity, (+) Retirement planning, (+) Crisis preparedness for difficult period immediately after retirement, (+) Support network, IMPACT OF **retirement on health/ wellbeing outcomes:** (-) Depression and pain |
| Athletes' career transition out of sport: a systematic review | Park, 2013 | Internat’l review of sport and exercise psych | Systematic review | 126 | Cross Sectional (113), Quantitative (56), Qualitative (55), Mixed (15), Longitudinal (13) | North America (61), Europe (47), Australia (10), Other (9). | **Gender:** combined gender (56), male (38), female (24) // **Level:** Elite (50), College or High School (32), Professional (27), Club (7)// **Sport Type:** Combined types (59), Team sport (36) Individual (26)// **Ages (at retirement):** 16-26 (53), 27-40 (21), wide range (17). | **Barriers/ facilitators of athlete transition**: (-)Athletic Identity , (+/-) Demographics, (+) Voluntariness of Decision, (-) Injuries/Health Issues, (+) Career development, (+) Sport achievement, (+) Education, (+) Financial, (+) Self-Perception, (+) Control over life, (+), Time passed since retirement, (+) Relationships with coach, (-)Life changes at time of retirement, (+) Life Balance while Competing. (+/-) Coping strategies, (+) Pre-retirement planning, (+) Psychosocial support, (+) Support program involvement |
| The sporting trajectory of elite athletes in artistic gymnastics: a systematic review | Vargas, 2021 | Science of gymnastics journal | Systematic review | 15 | All qualitative (15) | Brazil (7), Portugal (2), Sweden (1), Slovenia (1), Australian (2), England (1), New Zealand (1), International (1) | **Gender:** Female (12), Other (3)// **Level:** Elite clubs (12), Olympics (3)// **Sport:** All gymnastics// **Age of retirement:** Not reported | **Barriers/ facilitators of athlete transition:** (+) Seeking other professional paths while still in sports, (+) Reduce training in a gradual manner, (+) Planning for retirement |
| **REVIEWS ABOUT: Concussion, Cognitive, and Mental Health Implications of Athletic Retirement (n=8)** | | | | | | | | |
| History of Sport-Related Concussion and Long-Term Clinical Cognitive Health Outcomes in Retired Athletes: A Systematic Review | Cunningham, 2020 | J Athl Train | Systematic review | 46 | All quantitative (46) | Not reported in original text. | **Gender:** Male (43), Female (3)// **Level:** Not reported// **Sports:** American football (20), Rugby (7), Soccer (4), Boxers (3), Other (12)// **Age of retirement:** Not reported | **Barriers/ facilitators of athlete transition:** (-) History of concussion is a risk factor for poorer self-reported cognitive abilities (memory, executive function, psychomotor) |
| Influence of playing rugby on long-term brain health following retirement: a systematic review and narrative synthesis. | Cunningham, 2018 | BMJ Open Sport Exerc Med | Systematic review (and narrative synthesis) | 6 | All quantitative | Not reported in original text. | Not reported in original text. | **Barriers/ facilitators of athlete transition:** (-/+) Mixed findings of whether history of concussion leads to poorer self-reported cognitive abilities (memory, executive function, psychomotor) |
| Occurrence of mental health symptoms and disorders in current and former elite athletes: a systematic review and meta-analysis | Gouttebarge, 2019 | British Journal of Sports Medicine | Systematic review (and meta-analysis) | 34 | All quantitative | Not reported in original text. | Not reported in original text. | **Impact of retirement on health/ wellbeing outcomes**: In current athletes the prevalence of mental health symptoms ranged from 19% for alcohol misuse to 34% for anxiety and depression. In retired athletes this ranged from 16% for alcohol misuse to 26% for anxiety and depression. **Both are slightly higher than the general population. Barriers/ facilitators of athlete transition:** (+) Preparation for retirement, (+) Exit health examination |
| A systematic review of potential long-term effects of sport-related concussion | Manley, 2017 | Br J Sports Med | Systematic review | 47 | All quantitative | Not reported in original text. | Not reported in original text. | **Barriers/ facilitators of athlete transition**: (-) History of multiple concussions appears to be a risk factor for cognitive impairment and mental health problems |
| Prevalence and Correlates of Psychological Distress among Retired Elite Athletes: A Systematic Review | Mannes, 2019 | Int Rev Sport Exerc Psychol | Systematic review | 40 | All quantitative | US (21), Other (19)… Australia, Finland, Sweden, the United Kingdom, Ireland, France, South Africa, Germany, Belgium, Japan, Switzerland, Norway, France, Chile, Paraguay, Peru, and Spain | **Gender:** Male (32) Mixed (7), Female (1)// **Level:** Professional (24)/ Collegiate (6), International or Olympic (10**)// Sport:** Mixed (16), Soccer (6), American Football (14), Hockey (1), Rugby (3)// **Age of retirement:** 16-26 (2), 27-40 (14), not reported (24). | **Impact of retirement on health/ wellbeing outcomes:** (+/-) The prevalence of psychological distress among retired athletes is similar to that found in the general population. **Barriers/ facilitators of athlete transition:** (-) medical comorbidities, (-) significant pain, (-) a greater number of concussions, (-) less social support, and (-) adverse psychosocial factors were at greater risk for psychological distress after retirement. |
| Sport-Related Concussion and Mental Health Outcomes in Elite Athletes: A Systematic Review | Rice, 2018 | Sports medicine | Systematic review | 27 | All quantitative | USA (23), Canada (3), France (1) | **Gender:** Not reported// **Level:** Professional (7), College (19), Elite (1)// **Sport:** Mixed (15), American Football (10), Rugby (1), Hockey (1); **Age at retirement:** Not reported | **Impact of retirement on health/ wellbeing outcomes:** (-) There was some evidence of longer-term effects for elevated depression symptoms in retired athletes who experienced concussion. |
| Neuropsychological functioning in ageing retired NFL players: a critical review | Schaffert, 2020 | Int Rev Psychiatry | Critical review | 22 | All quantitative | Not reported in original text. | Not reported in original text. | **Impact of retirement on health/ wellbeing outcomes:** (-) There was some evidence to suggest that NFL retirees have a higher prevalence of cognitive impairment, suggesting retirees may be at greater risk for cognitive decline. |
| Long-Term Cognitive Performance of Retired Athletes with Sport-Related Concussion: A Systematic Review and Meta-Analysis. | Zhang, 2019 | Brain Sci | Systematic review (and meta-analysis) | 11 | All quantitative | Not reported in original text. | Not reported in original text. | **Impact of retirement on health/ wellbeing outcomes**: (-) The retired athletes who suffered from sports related concussions during their playing career had declined cognitive performance in partial domains (immediate recall, visuospatial ability, and reaction time) later in life. |
| **REVIEWS ABOUT: Musculoskeletal and Cardiovascular Implications of Athletic Retirement (n=5)** | | | | | | | | |
| Prevalence of osteoarthritis in former elite athletes: a systematic overview of the recent literature | Gouttebarge, 2015 | Rheumatol Int | Systematic review | 15 | All quantitative | Finland (7), Argentina (1), Sweden(2), Germany (4), Iran (1) | Gender: Male (15) Mixed (1)// Level: Not reported// Sport: Running/ Track and Field (6), Weight lifting (1), Shooting (2), Power sports (1), Tennis (1), Skiing (1), Mixed (2), Table Tennis (1).// Age of Retirement: Not reported. | **Impact of retirement on health/ wellbeing outcomes:**(-)This study showed that prevalence of OA, especially in their lower limbs, seems to be high among former elite athletes from team and individual sports compared to the general population and other occupational sectors. |
| Knee and ankle osteoarthritis in former elite soccer players: a systematic review of the recent literature. | Kuijt, 2012 | J Sci Med Sport | Systematic review | 4 | All quantitative | UK, (2), Tunisia (1), Slovenia (1) | Gender: Not reported// Level: All professional// Sport: Soccer// Age of retirement: Not reported | **Impact of retirement on health/ wellbeing outcomes**: (-)The prevalence of knee and ankle OA in former elite soccer players can be considered high compared to the general population and to other occupations. |
| Prevalence of knee osteoarthritis in former athletes: a systematic review with meta-analysis | Madaleno, 2018 | Braz J Phys Ther | Systematic review (and meta analysis) | 15 | All quantitative | International (1), UK (3), Tunisia (1), Greece (2), Finland (3), Denmark (1), Slovenia (1), Iran (1), Sweden (2) | Gender: Male (14), Female (1)// Level: Not recorded// Sport: Soccer (8), Mixed (4), Track and Field/running (2), Table tennis (1)Age of Retirement: Not reported., | **Impact of retirement on health/ wellbeing outcomes:** (-)Prevalence of knee osteoarthritis in former athletes was 30.0%. Previous studies showed that the prevalence of knee OA in general population ranged from 19.0 to 28.0%.40, 55 Thus, the prevalence of knee OA in former athletes may be higher than in general population |
| Cardiovascular Health of Retired Field-Based Athletes: A Systematic Review and Meta-analysis | McHugh, 2019 | Orthop J Sports Med | Systematic review (and meta analysis) | 13 | All quantitative | USA (12), Greece (1) | Gender: Male (13)// Level: Professional// Sport: American Football (10), Soccer (1)// Age of retirement: Not reported | **Impact of retirement on health/ wellbeing outcomes:** (+/-) Retired athletes had a comparable CVD risk profile with the general population. Retired athletes with an elevated body mass index had an increased prevalence and severity of risk factors. |
| Osteoarthritis of the hip and knee in former male professional soccer players | Petrillo, 2018 | British Medical Bulletin | Systematic review | 17 | All quantitative | Not reported in original text. | Gender: All Male// Level: All professional// Sport: All soccer// Age of retirement: Not reported | **Impact of retirement on health/ wellbeing outcomes:** (-) In fPSa, the prevalence rate of OA of both hip and knee is significantly higher compared to age and sex matched controls. |
