# Supplementary material for: Adaptation to life after sport for retired athletes: A scoping review of existing reviews and programs
Source: PLoS One. 2023 Sep 21;18(9):e0291683. doi: 10.1371/journal.pone.0291683 (PMC10513329; doi:10.1371/journal.pone.0291683)
Supplement: S3 Table — (DOCX) [file pone.0291683.s004.docx]

**Supplementary Appendix 3. Gray Literature Articles**

| **Title of article** | **Organization** | **Location** | **URL** |
| --- | --- | --- | --- |
| Life After Sport: Depression in Retired Athletes | Believe Platform | UK | <https://believeperform.com/life-after-sport-depression-in-retired-athletes/> |
| Mental Health Considerations of the Athlete Transition out of Sport | The Sport Information Resource Centre | Canada | <https://sirc.ca/blog/athlete-transition-out-of-sport/> |
| Preparing for Transition | Athlete Transitions | Canada | <https://athletetransitions.ca/preparing-for-transition> |
| How to Manage your Transition Out of Sport | Athlete365 | Global | <https://olympics.com/athlete365/career/how-to-manage-your-transition-out-of-sport/> |
| Athlete Transition: WHEN ALL YOU HAVE IS YOUR SPORT? | Athlete Assessments- Performance Through People | Australia | <https://www.athleteassessments.com/athlete-transition-when-all-you-have-is-your-sport/> |
| How to Support Athletes Who Transition Out of Sports | Northwestern- The Family Institute | USA | <https://counseling.northwestern.edu/blog/supporting-athletes-transitioning-out-of-sports/> |
| Sport Career Transition: Strengths, Challenges and Innovations Over Time | CERIC- Advancing Career Development in Canada | Canada | <https://ceric.ca/2017/02/sport-career-transition-strengths-challenges-and-innovations-over-time/> |
| What Professional Sports Can Teach Us About Career Transitions | Forbes | Global | <https://www.forbes.com/sites/pavelkrapivin/2019/10/30/what-professional-sports-can-teach-us-about-career-transitions/?sh=5a0576d77a05> |
| Career Transition | iresearch.net- Psychology Research and Reference | Global | <http://psychology.iresearchnet.com/sports-psychology/sports-psychology-careers/career-transition/> |
| Sport Retirement: What happens when the game's over? | Reaching Ahead | USA | <https://reachingahead.com/sport-retirement/> |
| Why Athlete Retirement Transitions Can be So Devastating | Front Office Sports | Global | <https://frontofficesports.com/athlete-retirement-psychology/> |
| Life After Retirement: The Difficult Transition For Athletes | Bruin Sports Business Association | USA | <https://www.uclabsba.com/articles/life-after-retirement-the-difficult-transition-for-athletes/> |
| Stepping Off The Field: How To Handle Retirement From Sports & Competition | GMTM | Global | <https://gmtm.com/articles/transitioning-from-sports-is-a-complex-process-not-to-beunderestimated> |
| Coping with Athlete Retirement | Trine University | USA | <https://www.trine.edu/academics/centers/center-for-sports-studies/blog/2021/coping_with_athlete_retirement.aspx> |
| Retirement, or Reinvention? | World Athletics | Sweden | <https://worldathletics.org/be-active/lifestyle/retirement-or-reinvention> |
| The Several Stages of Athlete Retirement | Athlete Career Transition | Global | <https://www.athletecareertransition.com/post/the-several-stages-of-athlete-retirement> |
| Retiring Athletes: The Road Ahead for the Retired Athlete | The Better You Institute | USA | <https://thebetteryouinstitute.com/2021/08/20/retiring-athletes/> |
| ‘A Big Struggle’: In Retirement, Athletes Can Face a Jarring Physical Transition | Global Sport Matters (Global Sport Institute at Arizona State University) | Global | <https://globalsportmatters.com/health/2021/09/07/retired-athletes-can-face-jarring-physical-transition-antone-davis-nfl/> |
| Professional Athletes Need a Retirement Game Plan | RBC Wealth Management | Global | <https://www.rbcwealthmanagement.com/en-us/insights/professional-athletes-need-a-retirement-game-plan> |
| Team Canada Athletes on Transitioning from the Sports World to the Business World | Canadian Olympic Committee | Canada | <https://olympic.ca/2020/11/09/team-canada-athletes-on-transitioning-from-the-sports-world-to-the-business-world/> |
| Top Mental Health Risk Factors for Retired Athletes | Purpose Sole Athletics | USA | <https://purposesoulathletics.com/top-mental-health-risk-factors-for-retired-athletes/> |
| Retired Athletes: The Lasting Effects Past the Sport | ScoopEmpire | UK | <https://scoopempire.com/retired-athletes-the-lasting-effects-past-the-sport/> |
| Life of the Retired Athlete: How to Fight Depression and Anxiety | Anchor Therapy LLC. | USA | <https://www.anchortherapy.org/blog/retired-professional-athlete-anxiety-depression-mental-health-hoboken-jerseycity-hudson-county-nj-therapist-counselor> |
| After Olympic Glory, Retiring Athletes Often Need Help Finding Their Way | CBC News | Canada | <https://www.cbc.ca/news/canada/olympics-athletes-retirement-1.3735834> |
| Transition to Retirement can Bring Emotional Turmoil for Elite Athletes | The Hamilton Spectator | Canada | <https://www.thespec.com/sports/olympics/2016/06/16/transition-to-retirement-can-bring-emotional-turmoil-for-elite-athletes.html> |
| Retirement Can be Scary for Olympians | Sportsnet | Canada | <https://www.sportsnet.ca/olympics/retirement-olympians/> |
| NOW WHAT AM I? WHEN IT’S GAME OVER FOR ATHLETES | CERIC- Advancing Career Development in Canada | Canada | <https://ceric.ca/2013/10/now-what-am-i-when-its-game-over-for-athletes/> |
| Retirement- What's your Game Plan? | Bank of Montreal (Wealth Institute) | Canada | <https://nesbittburns.bmo.com/getimage.asp?content_id=76712> |
| How Queen's University Prepares Olympians to Compete in Business | Maclean's | Canada | <https://www.macleans.ca/education/olympian-program-queens-university/> |
| Former Olympic Swimmer Mike Brown Talks Transition to Corporate Lawyer | The Kingston Whig Standard | Canada | <https://www.thewhig.com/business-sponsored/former-olympic-swimmer-mike-brown-talks-transition-to-corporate-lawyer> |
| Life After the Olympics | University of Calgary | Canada | <https://explore.ucalgary.ca/life-after-olympics> |
| Olympian Turned Journalism Grad Finds a Home on the Other Side of the Microphone | Carleton University Newsroom | Canada | <https://newsroom.carleton.ca/story/olympian-turned-journalism-grad/> |
| The Long Row Home: Athletes Call for More Post-Olympic Support | TVO Today | Canada | <https://www.tvo.org/article/the-long-row-home-athletes-call-for-more-post-olympic-support> |
| How to Transition After your Athletic Career Ends | Stack | USA | <https://www.stack.com/a/how-to-transition-after-your-athletic-career-ends/> |
| Post-Sport Transition Resources | Athlete Transition Study | USA | <https://www.athletetransitionstudy.com/resources/> |
| How Do Retired Athletes Transition to the World of Work? | Jason Hanold | USA | <https://jasonhanoldhr.com/how-do-retired-athletes-transition-to-the-world-of-work/> |
| When the Playing Days End | NCAA Champion Magazine | USA | <http://s3.amazonaws.com/static.ncaa.org/static/champion/when-the-playing-days-end/index.html> |
| Life After Sports | Athletes Connected (University of Michigan) | USA | <https://athletesconnected.umich.edu/for-student-athletes/life-after-sports/> |
| The Top 5 Reasons Why Athletes Struggle During Sport Retirement | The Sports Doc Chalk Chat | USA | <https://drstankovich.com/the-top-5-reasons-why-athletes-struggle-during-sport-retirement/> |
| Helping Student-Athletes Transition to Life After Sports | Unlock The Champion | USA | <https://www.unlockthechampion.com/our-tips-to-help-student-athletes-transition-to-life-after-sports/uncategorized/> |
| Many Retired Athletes Struggle with 'Unravelling of Identity' After Career Ends | CBC News | Canada | <https://www.cbc.ca/sports/athlete-post-retirement-life-identity-1.6585516> |
| Life After Sports: Why Athletes Need to Prepare | Athlete 365 | Global | <https://olympics.com/athlete365/whitepaper/life-after-sport-why-athletes-need-to-prepare/> |
| So What's Next?' - Life After Professional Sport | Believe Perform | UK | <https://believeperform.com/so-whats-next-life-after-professional-sport/> |
| Retirement from Sport | Sportweb | Canada | <http://www.sportweb.ca/Content/Athlete%20Handbook/Life/Transitions/Retirement%20from%20Sport.asp?langid=1> |
