# Supplementary material for: Adaptation to life after sport for retired athletes: A scoping review of existing reviews and programs
Source: PLoS One. 2023 Sep 21;18(9):e0291683. doi: 10.1371/journal.pone.0291683 (PMC10513329; doi:10.1371/journal.pone.0291683)
Supplement: S4 Table — (DOCX) [file pone.0291683.s005.docx]

**Supplementary Appendix 4. Gray Literature Programs**

| **Title** | **Organization** | **Location** | **URL** |
| --- | --- | --- | --- |
| Athlete Career Transition | Athlete Career Transition | Global | [https://www.athletecareertransition.com](https://www.athletecareertransition.com/) |
| Athlete Transitions | Athlete Transitions | Canada | [https://athletetransitions.ca](https://athletetransitions.ca/) |
| Game Plan | Canada Olympic Committee, the Canadian Paralympic Committee, Sport Canada and the Canadian Sport Institute Network | Canada | <https://mygameplan.ca/news/introducing-gameplan-canadas-athlete-career-transition-program> |
| Success after Sports | Success after Sports | Canada | <https://melinda-harrison-s-school.teachable.com/p/success-after-sport121> |
| YouToi2.0 | YouToi2.0 | Canada | <https://mygameplan.ca/news/introducing-gameplan-canadas-athlete-career-transition-program> |
| The Athlete's Journey | Global Sport Matters (Global Sport Institute at Arizona State University) | Global | <https://globalsportmatters.com/issue/athletes-journey/> |
| Transition Coaching | Athletes Soul | USA | <https://www.athletessoul.org/transition-coaching.html> |
| Athlete Career Transition Services | Nate Leslie- Certified Executive Coach | Canada | <https://nateleslie.ca/athlete-career-transition-services/> |
| Life After Sports- Athlete Transition & Retirement | Natalie Allport | Canada | <https://natalieallport.com/life-after-sports/> |
| 1-2-1 Support: Athlete Transition & Retirement | L&M Performance Psychology for Business and Sport | UK | <https://www.landmconsulting.co.uk/individual-support/athlete-transition-retirement-supportife-change-support/> |
| "Transforming the Arena" Athlete Transition Coaching Program | Habits for Wellbeing | Australia | <https://www.habitsforwellbeing.com/athlete-transition-coaching-program/> |
| Athlete Career Transition Programme | Curriculo Solutions | UK | <https://www.curriculosolutions.com/sport> |
| Athlete Mental Health & Sport Performance Counselling | Actualize Psychological Services | Canada | <https://actualizepsych.ca/services/individual-sport-performance/> |
| Moving On! | [athletesmovingon.org](http://athletesmovingon.org/) | USA | [https://athletesmovingon.org](https://athletesmovingon.org/) |
| Reboot | Reboot | South Africa | [https://www.rebootforlife.io](https://www.rebootforlife.io/) |
