# Supplementary material for: Adaptation to life after sport for retired athletes: A scoping review of existing reviews and programs
Source: PLoS One. 2023 Sep 21;18(9):e0291683. doi: 10.1371/journal.pone.0291683 (PMC10513329; doi:10.1371/journal.pone.0291683)
Supplement: S5 Table — (DOCX) [file pone.0291683.s006.docx]

**Supplementary Appendix 5. Stepwise Programming Content**

- **Step 1: Coming to terms with transition and seeing the retirement transition as a process:** *Understanding that there are aspects of your life that will no longer be the same. It’s important to process and make meaning from the end of the chapter, including processing any abuse or injury.*
  - **Detrain mind** **and body** – rely on coach, consultants, dieticians, strength personnel, consider a deceleration plan to slowly wean off of sports.
  - **Self-reflection questions** – acceptance of career ending, how they have found a sense of purpose and how they can apply learned skills to new opportunities.
  - **Committing to process** - taking time to transition and that retiring may be a long process, it takes time dedication and hard work.
  - **Taking time to pause** – time may be needed and take care of yourself before you decide what to do next, embrace trial and error to find the right next team.
- **Step 2: Understanding your identity and how your skills/strengths can transfer into a new setting:** *Understanding yourself and your current identity. Reflect on possible changes or transitions to this identity moving forward.*
  - **Recognize unique skills you have –** what do you really like about yourself and your current situation.
  - **Reduce exclusive identification with sporting role** – work to find interests outside of sport early, expand identity into other pursuits.
    - Develop new competencies in other activities.
    - Find purpose in day-to-day life.
    - Maintain commitment and regulation with other activities.
    - Develop a daily routine and engage with purpose and direction.
  - **Identify skill transferability** - like work ethic, planning, dedication, communication, leadership, relationships, resiliency, teamwork, discipline, time management.
  - **Identify values and characteristics** – what you liked and honed during sports, focus on assets that have helped succeed so far and how those can be tapped into, similarities between athletes and entrepreneurs.
  - **Identifying tangible proof** - identifying other ways you have already adapted these skills and shown these abilities.
  - **Identifying ways to continue in new ways** - continuing to train and keeping busy with a routine, positive reinterpretation, and thinking about all the great things you can take with you moving forward.
  - **Embrace that you are a life-long athlete** - the skills you have gained don’t just disappear.
  - **Reframing identity with sport** - thinking about ways to give back – how can you contribute to sport in new ways - Giving back through coaching and mentoring.
  - **Organizations can help –** they can find ways to keep and support retired athletes with the sports system, though coaching, mentoring and other options.
- **Step 3 - Gaining control over the change process and preparing for the future:** *Adjust to life without sports, and gain a broader sense of yourself and the new opportunities in front of you (personal, social, vocational, educational, physical.*
  - **Coping planning/ personal preparedness**
    - **Building resilience in other avenues outside of athletics.**
      - Recognizing resilience needed in athletic success and how this can be used during the transition navigation.
    - **Time management shifting.**
      - Pre-planning for how to fill time in a productive way.
    - **Coping resources.**
      - Identify resources, internal and external, to help with bumps along the way.
    - **Being aware of grief.**
      - Retirement can be a life crisis and may be associated with loss and grief.
      - Understanding that the road might be hard.
    - **Reflect on how you coped in the past.**
      - Reflect on previous transition experiences and successes.
      - Planning for back-ups (different career routes and options).
    - **Positive, ongoing self-evaluation and reflection.**
      - Optimistic framing.
      - Body scan and understanding daily emotional fluctuations, lean into emotions.
  - **Vocational/ educational future preparedness.**
    - **Prepare early, before transition happens.**
      - Investing more time in preparation, earlier.
      - Planning retirement leads to more success and enjoyment of sporting career.
      - Create a framework, structure your past, present, and future, and bridge the past, present, and future.
    - **Organizations should enable athlete transition preparedness.**
      - To self-manage their injuries and lifestyles outside of the system.
      - To enable open discussion about retirement.
      - Organizations should work with governing bodies to improve retirement support.
    - **Vocational planning.**
      - Do something that maintains high-self motivation and concentration, hinging on performance targets.
      - Building a professional network.
      - Not just about finding a job but a calling that you can go to.
      - Finding ways to achieve the natural high from competing.
    - **Educational planning.**
      - Continued education and learning.
  - **Social preparedness.**
    - **Establishing and maintaining personal relationships.**
      - With coaches, family, friends, partners, managers.
      - Prioritize communication with networks and rely on that robust support system.
      - Join new things with athletes already in your athletic network.
      - Finding a mentor.
    - **Establishing organizational and environmental relationships.**
      - With religion, occupational, community, physical settings etc.
    - **Find another team atmosphere.**
      - Create a community you can rely on.
    - **Knowing that you are not alone.**
      - Every athlete has gone through this or will go through it.
      - Sharing experiences with others.
      - Online platforms.
    - **Knowing connection is no longer mandatory.**
      - Making new friends may be more difficult.
    - **Organizations can help.**
      - Help family, friends, partners understand the athletic experience about the camaraderie, dressing room banter, travels with a team, routine of performance.
  - **Physical preparedness**
    - **Physical activity.**
      - Stay active and broaden sporting role to new activities.
      - Continuing to train and keeping busy with a routine.
      - Plan an exercise program to stay active.
      - Creating an ongoing training schedule.
      - Think about what other sports the athlete may enjoy and ease the transition.
    - **Body consciousness.**
      - Being aware of what your body needs and loving your body in new ways.
    - **Nutrition.**
      - Nutrition planning as sports training lessens.
  - **Financial preparedness.**
    - **Thinking about how to make money out of sports and identity.**
    - **Saving as early as possible.**
    - **Working with a financial advisor.**
- **Step 4: Normalizing the transition experience and living “in the next:”** *Building confidence in your new life directions, finding your sweet spot through trial and error, and developing a healthy self-identity with meaning outside of sport.*
  - **Athlete-led transition normalization.**
    - Understanding that you are not alone in your experiences.
    - Understanding what to expect in the retirement process.
    - Self-reflection and awareness of how transition is going.
  - **Sport-psychologist/ professional-led transition normalization.**
    - Building adaptation techniques.
    - Dealing with stages of grief and loss.
    - Set reasonable expectations based on your individual circumstance.
    - Personalized psychological support.
  - **Coach/team-led transition normalization.**
    - Gradual, early and integrated into team practices.
      - Encourage life outside of sport.
      - Find activities that give athlete purpose and new skillsets.
      - Help understand strengths as a person not just at performance.
        - Communication, leadership, relationships, resiliency, teamwork.
  - **Family/peer-led transition normalization.**
    - Ensure support groups are understanding.
    - Realizing that these other people might be affected as well.
